# An Overview of Candidate Therapeutic Target Genes in Ovarian Cancer

**DOI:** 10.3390/cancers12061470

**Published:** 2020-06-04

**Authors:** Elena Alexandrova, Giovanni Pecoraro, Assunta Sellitto, Viola Melone, Carlo Ferravante, Teresa Rocco, Anna Guacci, Giorgio Giurato, Giovanni Nassa, Francesca Rizzo, Alessandro Weisz, Roberta Tarallo

**Affiliations:** 1 Laboratory of Molecular Medicine and Genomics, Department of Medicine, Surgery and Dentistry “Scuola Medica Salernitan”, University of Salerno, 84081 Baronissi, Italy; ealexandrova@unisa.it (E.A.); gipecoraro@unisa.it (G.P.); assellitto@unisa.it (A.S.); vmelone@unisa.it (V.M.); cferravante@gmail.com (C.F.); trocco@unisa.it (T.R.); ggiurato@unisa.it (G.G.); gnassa@unisa.it (G.N.); frizzo@unisa.it (F.R.); 2 Genomix4Life, via S. Allende 43/L, 84081 Baronissi, Italy; aguacci@unisa.it; 3 CRGS-Genome Research Center for Health, University of Salerno Campus of Medicine, 84081 Baronissi, Italy

**Keywords:** ovarian cancer, molecular signature, fitness genes, hub molecules

## Abstract

Ovarian cancer (OC) shows the highest mortality rate among gynecological malignancies and, because of the absence of specific symptoms, it is frequently diagnosed at an advanced stage, mainly due to the lack of specific and early biomarkers, such as those based on cancer molecular signature identification. Indeed, although significant progress has been made toward improving the clinical outcome of other cancers, rates of mortality for OC are essentially unchanged since 1980, suggesting the need of new approaches to identify and characterize the molecular mechanisms underlying pathogenesis and progression of these malignancies. In addition, due to the low response rate and the high frequency of resistance to current treatments, emerging therapeutic strategies against OC focus on targeting single factors and pathways specifically involved in tumor growth and metastasis. To date, loss-of-function screenings are extensively applied to identify key drug targets in cancer, seeking for more effective, disease-tailored treatments to overcome lack of response or resistance to current therapies. We review here the information relative to essential genes and functional pathways recently discovered in OC, often strictly interconnected with each other and representing promising biomarkers and molecular targets to treat these malignancies.

## 1. Introduction

Ovarian cancer (OC) is one of the leading causes of cancer death in women, accounting for 295,414 new cases worldwide in 2018 and more than 180,000 victims [1]. According to the International Agency for Research on Cancer (IARC), the estimated global OC incidence for 2020 is 308,069 new cases and 193.811 deaths [2]. OC is an indolent disease, frequently diagnosed at advanced stages due to the lack of specific symptoms; current treatment of OC consists of surgery and systemic adjuvant or neoadjuvant chemotherapy; however, despite complete remission, the majority of initially responsive ovarian tumors often recur [3]. Given the poor prognosis of the disease, there is an urgent need to improve our knowledge of the genetic and molecular basis of OC to provide advances in the early detection and develop new treatment therapies.

Hormonal and reproductive factors are considered among the most significant risk factors for the development of OC. Early menarche or late menopause onset have been associated with a higher OC risk, suggesting that the ovulation-related proinflammatory response may promote malignant transformation and development of this gynecologic disease [4,5,6,7,8,9,10]. As a consequence, pregnancy, breastfeeding, and early menopause that preclude the ovulation, represent protective factors and can decrease the risk of OC developing [11,12,13,14]. Indeed, pregnancy, protects from OC through anovulation and decreased pituitary gonadotropins; multiple pregnancies confer up to 50% reduction of OC risk while nulliparity has been several times associated to ovarian carcinogenesis (29% increase of OC risk) [13,15,16,17]. In line with the anovulatory protective effect, breastfeeding has been inversely correlated to OC risk [18,19,20]. Literature also reports a consistent association between oral contraceptives assumption and reduction of OC risk, in particular when drugs are taken for a period ≥ 10 years [17,21,22,23]; particular effectiveness in risk prevention was observed when intrauterine contraceptives were used [24]. Conversely, the use of hormone replacement therapy (HRT) in post-menopausal women has been associated to an increased risk of ovarian cancer and it has been estimated that 55% of women who have used HRT, even for a short period, have developed OC during their lifetime [25].

According to the most probable tissue of origin, the World Health Organization groups ovarian tumors in surface epithelial cancers (65%), non-epithelial ovarian cancers including germ cell (15%) and sex cord-stromal tumors (10%), metastases (5%), and miscellaneous. Further classification of surface epithelial tumors takes into account cell type (serous, mucinous, endometrioid, clear cells), growth pattern (solid, cystic, surface), amount of fibrous stroma (cystadenoma and cystoadenofibroma) and atypia (benign, borderline, or malignant). Most of the malignant ovarian cancers are surface epithelial (90%) [26]; among them, the most represented ovarian cancer histotype is the serous (30%), followed by the mucinous (20%), endometrioid (15%), and clear cells carcinoma (5–10%) [27].

Few other rare types have also been described, such as Brenner tumors (malignant transitional cell tumor) and some mixed or undifferentiated carcinomas [28].

Lifestyle also affects the incidence of ovarian cancer; smoking, for example, has been associated with small increases in OC risk, in particular of the borderline mucinous type [29,30]. Other factors that negatively influence ovarian cancer onset include a high intake of saturated fats [31], high body mass index [32], and exposure to asbestos [33]. On the other hand, a moderate beneficial effect has been observed on ovarian cancer prevention in women that performed regular physical activity [34].

About 23% of ovarian cancer cases present a familiar inheritance pattern and are defined as hereditary neoplasms [35]; among them, 65–85% are caused by mutations in *BRCA1* and *BRCA2* genes, involved in the double-strand DNA breaks (DSBs) repair pathway, that cause 54% of OC lifetime risk increase. Other genes involved in DSBs repair and associated to hereditary ovarian cancer include *RAD51*, *PALB2*, *CHEK2*, *BARD1*, *Mre11*, *RAD50*, and *NBS1* [36]. Lynch syndrome, caused by mutations in mismatch repair (MMR) genes, also accounts for 10–15% of all hereditary ovarian carcinomas [37]. Lastly, there are genes related to other familiar cancer syndromes linked to an increased risk of ovarian cancer, such as *PTEN* (PTEN tumor hamartoma syndrome), *STK11* (Peutz-Jeghers syndrome) and *MUTYH* (MUTYH-associated polyposis) [38].

Genome-wide CRISPR-Cas9 dropout screening is emerging as a promising approach for characterization of driver genes of cancer growth [39,40]. Starting from the two main studies available so far, considering hundreds of human cancer cell models to identify essential genes for cell viability [41,42], in this review we deeply analyzed the data generated in a set of 48 OC cell lines in total, with the attempt to shed light on key molecular pathways involved and targetable in such an heterogeneous neoplasia. Focusing on 1213 essential genes in OC cells emerging from both studies, here we reviewed the functional pathways significantly affected by correlating computational information with experimental and, where possible, clinical data available in the literature. Finally, we also focused on the possible role of estrogen receptor-alpha (ERα), a debated candidate gene which is expressed in several OC histotypes, with high expression in serous ones [43]. Endocrine therapy has been used with modest and variable results in the treatment of OC [44], mainly due to tumor heterogeneity, thus it is not surprising that although crucial in OC progression, ERα did not emerge as a “fitness” gene within the investigated cell lines. Given the crucial role of this receptor in hormone-depending cancers and its relevance as therapeutic target, we aimed to extrapolate, among essential genes, those correlated with ERα activity and already implicated in relevant pathways for OC and that may be important for therapeutic purposes.

## 2. The Molecular Landscape of Ovarian Cancer

Advances in genomics technologies over the past decade have firmly established that, according to their molecular profiles, ovarian cancers can be classified into subtypes, each of them harboring distinct expression patterns, mutations, and epigenetic signatures. As a consequence, critical differences emerged between ovarian cancer subtypes in pathologic features, molecular changes, and clinical outcome; each subtype has been characterized, in fact, in its distinct genetic alterations, disease pathogenesis and progression, and survival outcome in response to therapy.

A recent classification ranks this heterogeneous group of malignancies into two broad categories: type I and type II. Type I ovarian cancers originate from clearly described primary ovarian lesions and comprise mucinous, endometrioid, low-grade serous, clear cell, and transitional cell carcinomas; on the other hand, the originating lesions of type II ovarian cancers are not well described and this category includes high-grade serous carcinomas, undifferentiated carcinomas, and carcinosarcomas [45].

Type I ovarian carcinomas generally develop from ovarian benign neoplasms, which in turn progress towards borderline and invasive carcinomas; they display a less aggressive behavior, with low metastatic spread at the time of diagnosis, stable genome, and generally no *TP53* mutations, although several somatic mutations have been described [46]. Type II ovarian carcinomas are generally more aggressive, diagnosed at advanced stages and with unstable genome, and frequently showing mutations in *TP53* or altered functions of *BRCA1/2* genes [47,48].

In the serous subtype of EOC two groups with different molecular profiles, clinical presentation and prognosis can be distinguished: the high-grade serous ovarian cancer (HGSOC), representing 90% of all serous tumors, and the low-grade serous ovarian cancer (LGSOC) accounting for the remaining 10% [49]. Compared with HGSOCs, associated to a poorer prognosis, usually diagnosed at late stages of the disease and frequently with metastases, the LGSOC group has a better prognosis and a significantly longer survival time [50]. Regarding their origin, HGSOC tends to originate in the fallopian tubes, spreading towards the ovaries and peritoneum, while LGSOC usually originates in the ovary [51,52].

Large-scale analyses combining expression profiles, mutational frequency and copy number alterations of HGSOC revealed a tendency in the upregulation of genes involved in chromosomal instability and cellular proliferation, providing evidence that defects in the homologous recombination DNA repair mechanisms (defective in >50% of cases) play a major role in the etiology of these tumors. Pathogenic somatic variants have been identified in genes involved in cell cycle regulation, DNA recombination, and DNA damage response and repair such as *TP53* (>95% of HGSOC), *FAT3*, *CSMD3*, *NF1*, *RAD51C*, *RAD51D*, *BRIP1*, *RB1*, *GABRA6*, *CDK12*, as well as germline and somatic mutations of *BRCA1* and *BRCA2* and loss of heterozygosity (LOH). Other genes frequently affected are *PTEN*, *RAD51C*, *ATM, ATR*, and many of the Fanconi anemia genes [28].

HGSOC are genomic-instable tumors with a tendency to copy-number variation resulting in the amplification or loss of several genes. More than 50% of HGSOC have homologous DNA repair pathway alterations and inactivation of tumor suppressor genes through gene breakage, mainly represented by *BRCA1* and *BRCA2*. Other characteristic genetic alterations (>20%) include *ID4*, *IRF2BP2*, *MYC*, *MECOM, PAX8, ZMYND8*, and *cyclin E1* (*CCNE1*) amplifications, the latter associated with resistance to therapy and poor prognosis; loss of *PTEN* is also predictive of poor prognosis. Signaling pathways frequently dysregulated include RB1, PI3K/Ras, Notch, and FOXM1 [28,53,54].

The molecular landscape of HGSOC reveals a strong tendency towards the variability of these tumors, whose genetic diversity promotes the development of distinct subclones, some of which may acquire pathogenic variants associated with resistance to the treatment and poor prognosis. On the other hand, the genomic instability of this subtype might also create variants that are more sensitive to chemotherapy, thus limiting cancer growth. For example, a better prognosis has been demonstrated for HGSOC in which genomic instability creates a higher response rate in platinum-based and poly ADP ribose polymerase (PARP) inhibition treatments [54].

Conversely to HGSOCs, LGSOCs are less aggressive, have a slower growth rate, and are genomic-stable. Expression profiling of LGSOCs has excluded the involvement of genes related to cell proliferation and DNA repair. Pathogenetic somatic variants of genes involved in signaling pathways are instead implicated, such as *KRAS*, *NRAS*, *BRAF*, *ERBB2*, and *PI3KCA* oncogenes. These alterations are accomplished by the frequent hyper-activation of the mitogen-activated protein kinase (MAPK) pathway, currently considered as a reasonable therapeutic target for LGSOCs, which have a poor response to the conventional chemotherapy due to the more competent DNA repair pathways [53,54].

Endometrioid ovarian cancers (EOVC) account for 10–20% of all EOC [55]. EOVC is a distinct and heterogeneous group of EOC; like serous tumors, both high and low-grade subtypes can be distinguished, with high-grade endometrioid tumors being very similar to HGSOC for their genomic instability and response to chemotherapy. Genomic profiling of endometrioid tumors has identified frequent activating mutations in *ARID1A*, *CTNNB1*, *KMT2D*, *KMT2B*, *PIK3CA*, *PTEN*, *PP2R1A*, and less frequently in *KRAS* and *BRAF* genes. Microsatellite instability, resulting from mismatch repair (MMR) deficiency and POLE mutation, was also observed [54,56].

Clear cell ovarian cancer (CCOC) comprises 5–10% of post-menopausal EOC and is characterized by a higher incidence among Asian women. Despite usually diagnosed at an early stage, CCOCs are less responsive to the platinum-based chemotherapy and have poor prognosis at late stages (III–IV) with respect to serous and endometrioid tumors [54,57].

Expression profiling has demonstrated that, from a molecular point of view, CCOC is more similar to lung cancer, endometriosis, and renal carcinoma than to other ovarian cancers [54,58,59,60,61]. The most frequently mutated genes in CCOC are *ARID1A* (46–57% of cases), *CTNNB1*, *CREBBP*, *KRAS*, *MLH1*, *PIK3CA*, *PPP2R1A*, and *PTEN* while a lower frequency of mutation is reported for BRCA1, BRCA2, and TP53 [54,62]. Genomic analyses have shown that CCOCs have mutations related to the process of ageing due to spontaneous deamination of methylated cytosines to thymines and leading to C-to-T mutations after DNA replication or misrepair of the DNA. Furthermore, CCOCs (>25%) exhibit a strong APOBEC signature, indicating alteration of genes encoding for enzymes involved in cytosine to uracile deamination [57].

Mucinous ovarian cancers (MOC) are rare when compared to other subtypes and comprise approximately 3% of EOC [63]. This heterogeneous subgroup differs greatly from the other EOC, showing morphological and genomic characteristics that appear to be more closely related to colorectal cancer. Among the genetic abnormalities identified, it is worth mentioning a high frequency of somatic *KRAS* variants (also found in other ovarian cancer types) and *ERBB2* amplifications. Other molecular features distinctive of MOCs are mutations of *RAS* (45%) and *BRAF* (22.6%) and the overexpression of *HER2. TP53* mutations are also present in 51% of MOC and associated with an increasing degree of malignancy. Additional frequently mutated genes are *CDKN2A*, *CTNNB1*, *PIK3CA*, *PTEN*, and *RRAS2* [53,54].

## 3. The Role of Estrogen Receptors in Ovarian Cancer

Estrogens are a class of steroid hormones secreted by granulosa cells in the ovary; the predominant intracellular estrogen is 17β-estradiol (E_2_). Estrogens are involved in many physiological and pathological processes in the reproductive, cardiovascular, skeletal, endocrine, nervous, and immune systems. They are able to regulate the expression of several genes involved in cell development and proliferation and are known to be involved in breast carcinogenesis. In ovarian cancer, the role of estrogens is still debated but there is a growing body of evidence supporting the contribution of estrogens as risk factors after prolonged exposure to them [64]. Furthermore, there is some evidence supporting the involvement of the estrogen pathway in ovarian cancer progression [65,66]. Estrogen-mediated gene regulation occurs through two estrogen receptors (ERs), members of the nuclear receptor family of transcription factors, ERα and ERβ, encoded by ESR1 and ESR2 genes, respectively [67]. Once activated by estrogens, ERs dimerize and translocate to the nucleus where, after the recruitment of co-regulators, they directly bind to estrogen-response elements (ERE) on the genome and regulate the expression of target genes both positively and negatively [68].

In ovarian tissues, both ERα and ERβ are expressed [69]. In women of childbearing age, ERα is mainly located at theca cells, in the ovarian stroma, in the corpus luteum, and surface epithelium of the ovary. In postmenopausal women, ERα is expressed in the stroma, in the epithelial inclusion cyst, and in the ovarian-surface epithelium. The main locations of ERβ are granulosa cells [70]. In the development of ovarian cancer, ERs show different behavior: ERα shows pro-tumorigenic activities while ERβ acts as a tumor suppressor [43,71,72].

ERα is expressed in more than 50% of OCs and in approximately 80% of HGSOC, where its expression is associated with a poor prognosis [42,73,74]. ERα involvement in ovarian cancer progression is related to several processes, including cell proliferation induction, invasion and metastasis, and chemo-resistance. The binding of estrogens to ERα induces the transcription of genes that stimulate cell proliferation. It has been demonstrated that ERα can mediate mitogenic signaling activation, in OC cells, both in vivo and in vitro; this happens through the expression of several genes including *MYC*, *PGR,* and *IGFBP3* [43]. It is known that the Mitogen-Activated Protein Kinase (MAPK) signaling can interact with hormonal mediators, such as ERα in its non-genomic pathway. The MAPK signaling pathway is a major regulator of cell proliferation, survival, and differentiation. Hyperactivation of this pathway occurs in EOC via gain of function mutations in Ras or Raf, which is thought to promote neoplastic transformation [75]. Cell proliferation via ERα is also mediated by activation of the Akt, ERK, and PI3K cascades [76,77] in OC cells. O’Donnel et al. observed that ERα mediates both growth response and gene expression changes in ovarian cancer cells exposed to E_2_. Indeed, many ERα-regulated genes in ovarian cancer cells have been reported, such as regulators of the cell cycle (*CCNB1*), apoptosis (*TNFSF7, TRAP1, UBL1*, and *CASP4*), transcription (*FOSL1, TFAP4, EIF2B1*), signaling (*NOTCH4, IGFBP3*, *BENE, LCN2*, *GRSF1*), and modulators of cytoskeleton and extracellular matrix remodeling (*CTSD*, *CDH6, CYR61, KRTs* 4, 7, and 13, *VIM, TGFBI, DES, AKAP12, TRAM1, MMPs* 11 and 17, *PLAU*) that could be involved in invasion and metastasis [78]. It has been also reported that PAX2 is activated by E_2_ via ERα in breast cancer and it is confirmed that the expression of PAX2 is proportional to the expression of ERα in ovarian serous cancer [79].

Epigenetic mechanisms have emerged as contributing factors to carcinogenesis. A recent work from our group elucidated the role of the DOT1L (disruptor of telomeric silencing-1-like) gene as a regulator of ERα activity in estrogen-responsive OCs [80]. DOT1L, a histone methyl transferase, acts as transcriptional regulator through H3K79 mono-, di-, and tri-methylation. ERα cooperates with DOT1L to modulate, at transcriptional level, the expression of genes involved in OC cell proliferation and other key cellular functions. Indeed, ERα or DOT1L inhibition, with selective antagonists, results in a dose-dependent reduction of OC cell proliferation.

The involvement of ERα has also been described in the invasion and metastasis mechanisms in OC cells where E_2_ is able to increase the metastatic potential of human epithelial ovarian cancer cell lines and enhance cell migratory potential through an ERα-dependent pathway [81]. Furthermore, the involvement of ERα in the invasion mechanism through the activation of Plexin B1 was also observed. Plexin B1 is an oncogene involved in cell migration that is positively regulated by ERα and negatively regulated by ERβ [82].

Moreover, it has been observed that CXCR7 (C-X-C Chemokine Receptor Type 7) and CXCL11 (C-X-C motif chemokine 11) genes are activated by estrogens through the direct recruitment of ERα and this leads to increased migration and invasion of OC cells [83]. Estrogens are also able to influence the anoikis process by Bit1 involvement. Cancer cells are generally more resistant to anoikis and this contributes to metastasis and invasion. Bit1 (Bcl2-inhibitor of transcription 1) is a mitochondrial protein involved in the cell death machinery after its release from mitochondria. In the cytosol, Bit1 forms a complex with AES (a member of the Groucho family of transcriptional corepressors) and promotes apoptosis. E_2_-activated ERα, decreases Bit1 level in the cytosol, which determines anoikis reduction in OC cells [84].

Lastly, ERα can influence OC cell response to chemotherapeutic agents. ERα can be activated by cisplatin via ERK cascade activation through the phosphorylation at serine 118. This can induce platinum-resistance by increasing the expression of anti-apoptotic proteins like Bcl-2. Contrarily, ERα downregulation is able to inhibit cisplatin-resistance [76]. All this evidence supports the possibility that, although understudied, ERα represents an effective target in the treatment of OC even though resistant to conventional chemotherapeutic agents.

## 4. Genome-Wide CRISPR-Cas9 Dropout Screening for Identification of Candidate Therapeutic Target Genes in OC

High-throughput CRISPR-Cas9 functional genomic screenings have allowed to perform a genome-wide perturbation of gene expression and determine the involvement of specific genes in cellular processes, thus understanding the hub genes causing diseases and exploring the responsiveness and resistance to drugs. The most popular and simplest approach to characterize the genetic drivers of tumor growth is the dropout screening, which allows the identification of fitness genes, defined as context-dependent essential genes that regulate the proliferation and/or survival of cancer cells under specific growth conditions. This approach also enables the identification of genes that are essential in cancer but not in normal tissues and therefore represent optimal therapeutic targets with minimal side effects [39,40].

Several studies have pointed out the efficiency of high-throughput CRISPR/Cas9 screening in the identification of cancer-related genes in ovarian cancer. Kodama et al., in their work, performed an in vivo dropout screen in human tumor xenografts using a pooled shRNA library targeting thousands of druggable genes to find out a list of 10 potent drug targets for EOC, including the novel oncogene KPNB1 [85]. He et al. applied a loss-of-function CRISPR screen and recognized DYNLL1 as an inhibitor of DNA end resection, whose loss in BRCA1 deficient HSGOC cells induced resistance to platinum drugs and inhibitors of poly(ADP-ribose) polymerase [86]. Similarly, Fang et al. identified C12orf5, encoding TP53 induced glycolysis and apoptosis regulator (TIGAR), as a novel therapeutic target able to modulate ovarian cancer sensitivity to the PARP inhibitor olaparib [87]. In addition, Qianying et al. shed light on a group of genes involved in cisplatin resistance in ovarian cancer cells, identifying ZNF587B as a novel predictive marker [88], whereas Stover et al. performed a near genome CRISPR/Cas9 screen in BRCA2 mutant HGSOC cell lines and identified BCL2L1 as a gene that mediates resistance to platinum-based chemotherapy [89]. Overall, it is evident that the scientific community is widely focusing on the application of knockout loss-of-function screenings to identify novel exploitable targets in the constant search for effective drugs able to overcome the major problem of chemo-resistance.

Among the large-scale CRISPR-Cas9 dropout screenings generated so far, two independent studies have been performed across hundreds of human cancer cell lines at the Broad and Sanger Research Institutes [41,90,91]. Here, we collected genome-scale CRISPR-Cas9 screening data from the Achilles project at Broad Institute through the DepMap portal [92] and from Sanger Project Score [93]. In total, 48 ovarian cancer cell lines, representative of the main molecular subtypes of OC (Serous, Mucinous, Endometrioid, and Clear Cells) and some rare tumors, were taken into account (Table 1) and 18,333 and 17,995 genes were independently screened from Broad’s and Sanger’s datasets, respectively. The reduction of cell viability upon gene inactivation was quantified using individual gene scores across cell lines (gene dependency profiles) using fully processed gene scores available for download from the Broad and Sanger Cancer Dependency Map webportals. A gene was considered fitness if the CERES score was ≤ -0.5 for Broad’s data and Average Score ≤ 0 for Sanger’s data.

## 5. Functional Pathways Affected by OC Fitness Genes

By comparing the two datasets above mentioned, 1213 common fitness genes were identified (with 2034 and 1410 essential genes observed in Broad and Sanger studies, respectively).To elucidate the functional pathways connected to fitness genes in the pathological environment of OC, we performed a Gene Ontology (GO) analysis using the Ingenuity Pathway Analysis (IPA, QIAGEN, Redwood City, www.qiagen.com/ingenuity) tool. As a result, we obtained a distribution map of the OC fitness genes made of interconnected nodes across biological processes critical for survival and proliferation of malignant cells and for tumor growth. Crucial signaling pathways, whose alterations represent the hallmarks of cancer, were identified, including cell cycle regulation and DNA repair mechanisms, hypoxia and angiogenesis processes, proliferative signaling, RNA translation and post-translational modifications, protein degradation, nucleotide metabolism, etc. In Table 2, the 54 canonical pathways most significantly affected, together with the involved fitness genes, are reported.

### 5.1. DNA Damage Response Associated Pathways

**Figure 1:**
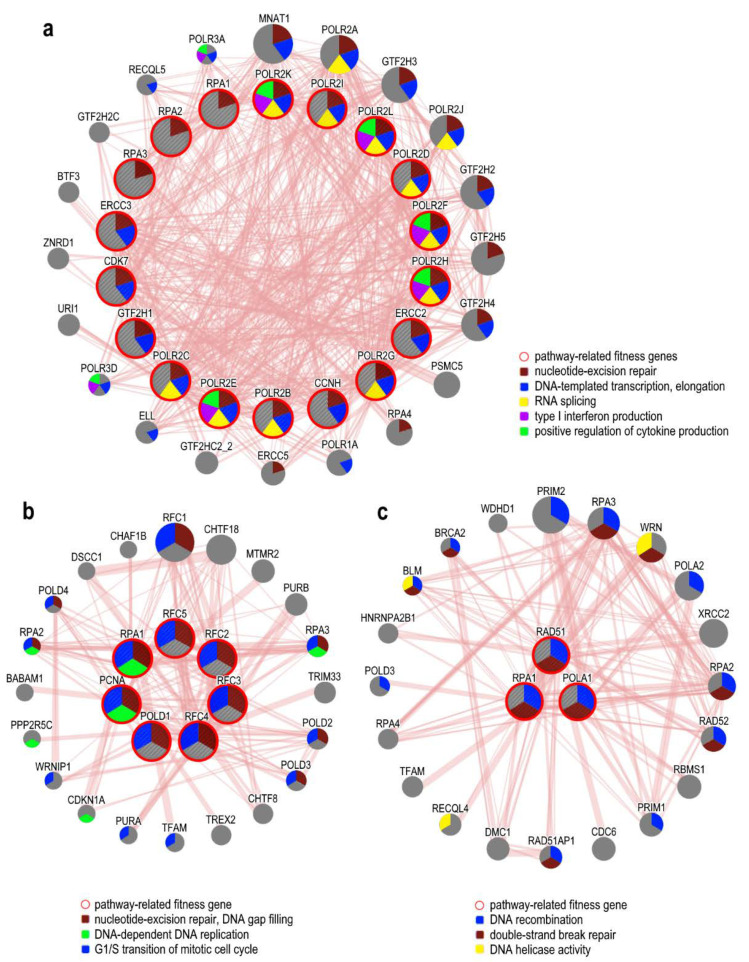
Network reconstruction analysis and functional enrichment of ovarian cancer fitness genes representative of DNA Damage Response (DDR) mechanisms, performed using GeneMANIA (genemania.org/). (**a**) Nucleotide excision repair (NER) mechanism. (**b**) DNA mismatch repair mechanism. (**c**) Homologous recombination (HR) mechanism. Protein–protein physical interactions between fitness genes in OC (internal shell) and related genes connected to them (external shell) are shown. Each node represents a gene and the edge width is proportional to the strength of the interactions. Gene association to biological processes is represented with a color code (legends).

Among the most-significant cancer-associated alterations, aberrations in the DNA damage response (DDR) play a major role in OCs. The constituent pathways of the DDR include DNA repair machinery, cell cycle checkpoints, and apoptotic pathways; mutations in any components of these pathways are involved in the ovarian cancer initiation and progression as well as in resistance to therapy [95]. Fitness genes identified by the dropout CRISPR-Cas9 screening in ovarian cancer cell lines include key-genes involved in the cell cycle checkpoint regulation, components of the mismatch repair (MMR) system that recognize and repair DNA abnormalities, members of the homologous recombination (HR) and nucleotide excision repair (NER) pathways (see Table 2 and Figure 1).

Alteration of regulatory mechanisms of the cell cycle results in uncontrolled cell proliferation which is a hallmark of cancer; these alterations occur in cyclins, cyclin-dependent kinases (CDK) and CDK inhibitors. In serous ovarian carcinoma, high expression of P16, P53, and P27 and low expression of P21 and cyclin E has been reported [96]. AURKA, CDC25, cyclin B and PLK1, have been reported to be overexpressed in OC [97]; furthermore, both AURKA and CHEK1 were associated with detrimental outcome in early-stage OC [98]. In this context, targeting of DNA repair mechanisms in combination with inhibition of key regulators of the mitotic process could be useful for ovarian cancer treatment. Indeed, a promising synergistic antitumoral effect between AURKA and CHEK1 inhibitors in ovarian cancer has been described [97]. WEE1 kinase, encoded by the fitness gene WEE1, is frequently expressed in ovarian serous carcinoma and plays a key-role in the G2 cell cycle checkpoint arrest for pre-mitotic DNA repair. Abrogation of the G2 checkpoint through WEE1 inhibition could result in increased antitumor activity of DNA damage-inducing chemotherapeutic agents. WEE1 expression is significantly higher following exposure to chemotherapeutic agents [99]; the combination of chemotherapy with WEE1 inhibitors is therefore particularly promising in ovarian cancers [100]. Replication factor C (RFC) also plays a crucial role in the checkpoint control of cell cycle progression. RCF3 subunit is overexpressed in OC and has a prognostic value in predicting patient survival. RCF3 knockdown has been demonstrated to reduce viability and proliferation in OVCAR-3 cells by blocking the cell cycle in the S-phase and inducing apoptosis, suggesting that it could be a potential target in the clinical practice [101].

Fitness genes identified in ovarian cancer cell lines also included many DDR transducers; among them, a critical role is played by ATR. Once activated by DNA damage, ATR blocks the cyclin-dependent kinases CDK1 and CDK2 (also identified among OC fitness genes) thus preventing cell cycle progression. Cyclin-dependent kinases CDK1 and CDK2 regulate the expression of other proteins involved in DNA repair, cell cycle control, and apoptosis; dysregulation of their activity is frequently associated with inappropriate cell-cycle progression. High ATR expression in ovarian cancer tissues has been linked with poor survival and progression free-survival, while it has been identified among the critical factors in determining platinum sensitivity in cell lines models [102]. A defective DNA-damage response (DDR) is a defining hallmark of high-grade serous ovarian cancer (HGSOC). In HGSOC cell lines, PARP inhibitors (e.g., olaparib) in combination with drugs targeting the ATR/CHK1 axis resulted in tumor regression in BRCA-mutant ovarian cancer [103]. ATR inhibitors can sensitize ovarian tumors to DNA-damaging agents that primarily induce replicative stress as their mechanism of action [104]. Several other fitness genes are interconnected in this pathway, including those encoding for HUS1, RAD1, and RAD9 proteins that form a hetero-trimer acting as a sensor of DNA lesions. HUS1 overexpression has been correlated with worst prognosis and with high expression of P53 and BAX and high mitotic and apoptotic indices in OC [105]. RAD1 and CHEK1, other fitness genes involved in cell cycle regulation, are crucial factors required for the check-point mediated cell cycle arrest and activation of DNA repair by homologous recombination (HR). The two genes have also been associated with a BRCA-like phenotype in hereditary breast and ovarian cancer. In OC cells, RAD1 and CHEK1 knockdown led to decreased cellular viability, increased sensitivity to cisplatin, and decreased HRR efficiency [106], while CHEK1 overexpression was associated with detrimental outcomes in early-stage ovarian cancer [98]. RAD9, a pro-apoptotic protein, has been associated with higher mitotic and apoptotic indices [105].

DNA double-strand break repair by homologous recombination (HR) uses DNA sequence homology and exploits genetic information available on an undamaged sister chromatid or homologous chromosome [107]. The HR process has its core in the nucleation of the RAD51 filament, which competes with the ssDNA-binding protein RPA, whose role is to protect single-strand DNA from degradation and formation of secondary structures that would interfere with repair. Once RAD51 nucleation prevails, the process of strand invasion in the unbroken identical DNA molecule begins and allows the repair mechanism to work properly [108]. Population studies have showed that deleterious mutations in RAD51 paralogs RAD51C and RAD51D confer susceptibility to epithelial ovarian cancer [109], while specific polymorphisms of the RAD51 gene could be used as a biomarker for increased risk of OC [110]. Moreover, RPA availability seems to be related to chemo-resistance in HGSOC [111].

Recently, the HR pathway has attracted considerable attention not only for its role in the repair of DNA damages induced by chemotherapeutic agents, but also because many cancers are, to different extents, defective in HR repair, raising the possibility to exploit this feature for novel cancer treatments. In this context the concept of synthetic lethality acquires high relevance, that leads to cell death when two otherwise non-lethal defects occur simultaneously and synergize; using inhibitors of poly(ADP-ribose) polymerase (PARP), a protein involved in DNA repair processes, it is in fact possible to kill specifically HR-deficient cancer cells [112,113].

The main pathway for the removal of large DNA lesions is instead the nucleotide excision repair (NER); this system is active towards single stranded DNA damages; the damaged strand is removed and the gap filled replaced by DNA synthesized using the undamaged strand, and the two ends are joined together by a DNA ligase. The NER system plays a key role in OC for its prognostic value and in response to treatment. Among fitness genes belonging to the NER pathway we identified some crucial factors, whose mutations are strongly correlated to cancerous phenotype, such as POLE, RPA3, and ERCC genes [95]. Excision repair cross-complementing DNA-helicases ERCC2 and ERCC3 belong to the transcription factor IIH complex and unwind DNA strands that flank the damaged site. Although ERCC2 has been correlated with a more aggressive phenotype in head and neck tumors, the role of both proteins in OC is still unclear [114]. A higher somatic mutation burden has been recently reported in OC for the POLE gene, encoding for an enzyme involved in the DNA repair and replication; the impact of POLE mutations also seems to be more prominent in sporadic OC than in familiar one [115]. Upregulation of the RPA3 gene has been associated with HGSOC proliferation [116].

MMR is critical for the detection of DNA damages as deficiency in this pathway could lead to uncontrolled proliferation. Loss of function in the hub genes of the MMR pathway have been identified in 29% of ovarian cancers and their mutations correlate with the neoplasm stage [95]. In Figure 1 some of the functional pathways specifically related to DNA damage repair mechanisms are reported as an example; in particular nucleotide excision repair (Figure 1a), DNA mismatch repair (Figure 1b), and homologous recombination (Figure 1c) have been depicted, with fitness genes specifically correlated and physical interactions among the different proteins involved in the pathways.

### 5.2. Hypoxia and Angiogenesis Related Genes

After evading growth suppressors and escaping apoptosis, cancer cells must face hypoxia and low nutrient levels, peculiar characteristics of the tumor microenvironment, to support their energy metabolism and sustain their growth. Therefore, it is not surprising that hypoxia-dependent signaling pathways are commonly de-regulated in cancer cells. A recent study demonstrated that clear cell and serous EOC are under constant endoplasmic reticulum (ER) stress caused by the accumulation of unfolded proteins; due to this, the unfolded protein response (UPR) sensor PERK located in the ER activates and phosphorylates the eukaryotic translation initiation factor eIF2, resulting in a general suppression of translational initiation and global protein synthesis [117].

**Figure 2:**
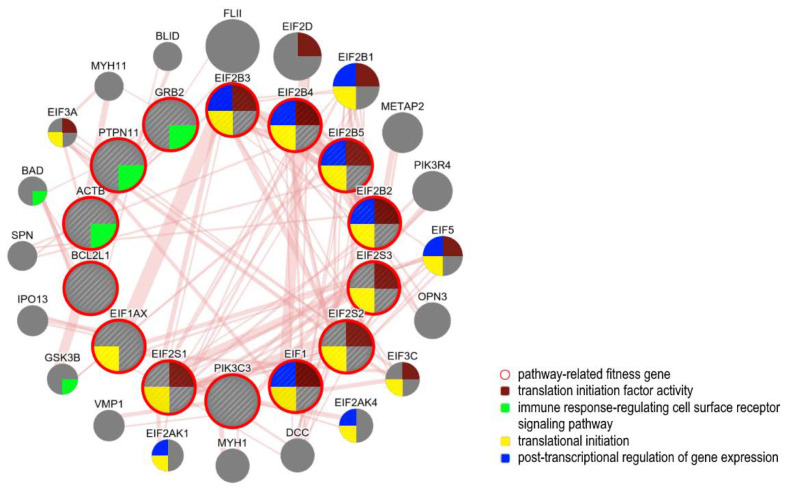
Network reconstruction analysis and functional enrichment of ovarian cancer fitness genes representative of the VEGF signaling, performed using GeneMANIA (genemania.org/). Protein–protein physical interactions between fitness genes in OC (internal shell) and related genes connected to them (external shell) are shown. Each node represents a gene and the edge width is proportional to the strength of interactions. Gene association to biological processes is represented with a color code (legend).

Interestingly, there are several lines of evidence suggesting that this mechanism is also linked to the estrogen signaling, since a role for eIF2α as a key regulator of estrogen-induced apoptosis has been recently demonstrated in estrogen sensitive MCF-7 breast cancer cells through PERK-mediated phosphorylation [118]. Tumor hypoxia is also a major regulator of the angiogenesis process, where new abnormal vasculature is formed around the tumor, thus providing nutrients to the malignant cells and supporting tumor growth. A key-mediator of angiogenesis in cancer is the VEGF, a cytokine whose expression is regulated by several factors including hypoxia; once activated, VEGF promotes endothelial cells proliferation, migration, and vascular permeability. Strongly implicated in normal ovarian function, VEGF plays a critical role in OCs, high-vascularized tumors; its overexpression represents an early event in ovarian carcinogenesis and is associated with tumor progression and poor prognosis [119,120]. In breast cancer cells, the expression of VEGF is induced by estrogens through the association of ERα to the estrogen response elements (EREs) located within the promoter region of the gene. On the other hand, in endometrial carcinoma cells, VEGF transcription is regulated by 17β-estradiol (E2) through a variant ERE localized ≈1.5 Kb upstream the VEGF transcription start site [120], thus indicating that the estrogens may directly regulate tumor angiogenesis also in ovarian cancer. Key genes involved in VEGF signaling, including OC fitness genes retrieved, are reported in Figure 2.

### 5.3. Proliferative Signals

One of the fundamental traits of cancer cells involves their ability to sustain proliferation by deregulating the normal cell growth and division cycle that ensure the homeostasis of cell number and the maintenance of normal tissue architecture and function. Mitogenic signals are mainly represented by growth factors that bind cell-surface or intracellular receptors stimulating cell proliferation.

**Figure 3:**
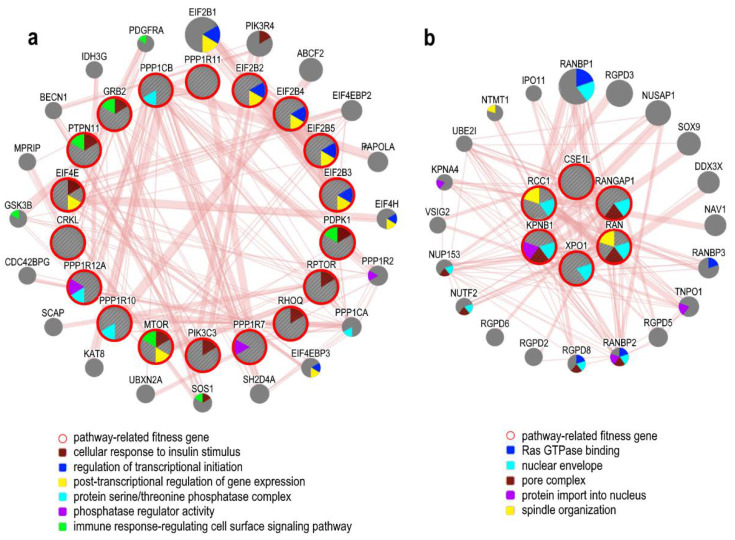
Network reconstruction analysis and functional enrichment of ovarian cancer fitness genes representative of proliferative signaling, performed using GeneMANIA (genemania.org/). (**a**) Insulin receptor related genes. (**b**) Ran signaling related genes. Protein–protein physical interactions between fitness genes in OC (internal shell) and related genes connected to them (external shell) are shown. Each node represents a gene and the edge width is proportional to the strength of interactions. Gene association to biological processes is represented with a color code (legend). Abbreviation: Ran, Ras-related Nuclear protein.

Among the fitness genes identified in the investigated OC cells, many take part in the insulin receptor pathway (Figure 3a). The insulin receptor can mediate a trophic effect in some transformed cells by activating mitogenic signals [121]. In many preclinical studies, the inhibition of insulin receptor shows a reduction in growth of ovarian cancer models and potentiates the efficacy of platinum-based chemotherapy. However, despite the pre-clinical data, anti-IR targeted strategies lacked efficacy in the clinic [122]. Mitogenic pathways activated by the insulin receptor are the canonical phosphatidylinositol 3-kinase (PI3K)-AKT, mTORC1, and RAS-extracellular signal-regulated kinase (ERK) pathways. Hyperactivation of these pathways is implicated in the development, maintenance, progression, and survival of ovarian cancer. PI3K, AKT, and mTOR, are highly mutated or overexpressed in a high percentage of ovarian cancer patients and are associated with advanced grade and stage disease and poor prognosis. This pathway could represent a target for OC therapies [123]. Moreover it has been noticed that the PI3K/Akt/mTOR signaling is required for E-cadherin downregulation and involved in the invasion mechanism [124]. Another pathway revealed by the IPA analysis is the Ran signaling (Figure 3b). Ran, a member of the Ras GTPase family, is a nucleocytoplasmic shuttle protein that is involved in cell cycle regulation, nuclear-cytoplasmic transport, and plays an important role in cancer cell survival and progression. This protein is highly expressed in epithelial ovarian cancers where it is associated with a poor prognosis [125]. It was seen that Ran downregulation induces caspase-3 associated apoptosis and causes a delay in the tumor growth. These results suggest that Ran could potentially be a suitable therapeutic target for OC treatment [126]. Moreover, other Ran-related factors are known to be involved in ovarian cancers, such as CSE1L, the human homolog of the yeast cse1gene. CSE1L is overexpressed in ovarian cancer where it is related to adverse patient outcomes. CSE1L forms a complex with Ran and importin-α and regulates nucleocytoplasmic traffic and gene expression. CSE1L protects ovarian cancer cells from death both in vitro and in vivo by suppressing the pro-apoptotic RASSF1 gene. The nuclear accumulation of CSE1L improves the expression of pro-oncogenic genes it regulates [127]. In addition to tumor growth, Ran is also involved in metastasis mechanisms. There is a link between Ran and RhoA signaling that contributes to enhanced ovarian cancer cell growth and invasiveness. RhoA is a Rho GTPase able to regulate many aspects of cell invasion and its expression is associated with advanced stage of ovarian cancer. Ran can form a complex with RhoA, leading to RhoA stabilization and activation. This Ran-RhoA signaling complex could be a molecular target for controlling cancer metastasis [128].

### 5.4. ER-Related Pathways and Fitness Genes in OC

**Figure 4:**
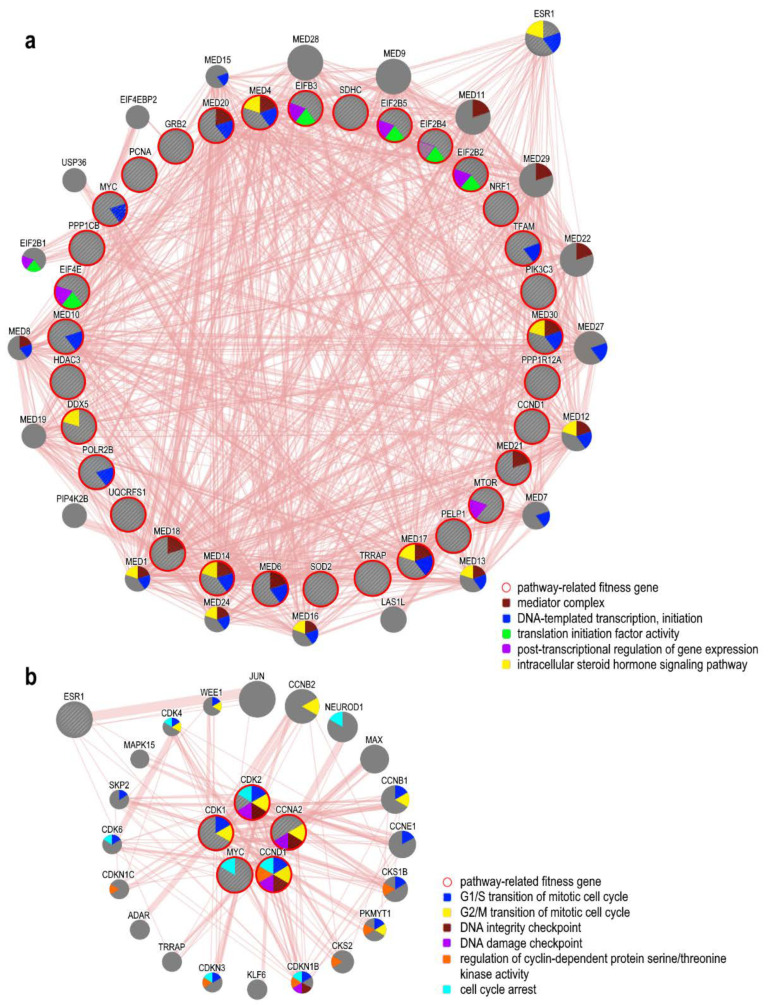
Network reconstruction analysis and functional enrichment of ovarian cancer fitness genes representative of the endoplasmic reticulum (ER)-related pathways, performed using GeneMANIA (genemania.org/). (**a**) Estrogen receptor signaling related genes. (**b**) Estrogen-mediated S-phase Entry related genes. Protein–protein physical interactions between fitness genes in OC (internal shell) and related genes connected to them (external shell) are shown. Each node represents a gene and the edge width is proportional to the strength of interactions. Gene association to biological processes is represented with a color code (legends). ESR1 has been inserted among the pathway-related genes to show fitness genes physically interacting with it.

The signaling of ERα mediates mitogenic activation in OC cells by regulating the expression of genes promoting cell proliferation [43]. Among the fitness genes identified by the CRISPR-Cas9 drop-out screening, 35 were directly involved in ER related pathways (see Table 2 and Figure 4), including the MYC oncogene, frequently amplified in OCs, MTOR, CCND1 CDK2 CCNA2 CDK, PCNA, PPP1R12A PIK3C3 EIF4E HDAC3, PPP1CB, involved in cell cycle regulation and cell proliferation, EIF2B3, EIF2B4, EIF2B2, EIF2B5, connected with the activation of the immune response, MED10, MED14, MED17, MED18, MED20, MED21, MED30, MED4, and MED6 members of the Mediator (MED) complex, an evolutionary conserved multiprotein involved in RNA polymerase II-dependent transcription, whose aberrations have been reported in several malignancies including OC [129].

Among others, we noticed the proliferating cell nuclear antigen (PCNA), a processivity factor for DNA polymerase δ, involved in the recruitment of DNA replication-related proteins [130]. It has been observed that PCNA expression can be positively regulated by ERα and correlates to increased cell proliferation and cell cycle progression; moreover, immunostaining assays to evaluate the presence of the protein can be applied to define different prognostic subgroups in ovarian cancer patients [131].

To further characterize the expression and mutational landscape of ERα-associated fitness genes, we explored three Ovarian Serous Cystadenocarcinoma cohorts (PanCancer, Nature 2011 and Firehose Legacy) collected by The Cancer Genome Atlas (TCGA) database, altogether comprising 1680 ovarian cancer tissues from 1668 patients.

**Figure 5:**
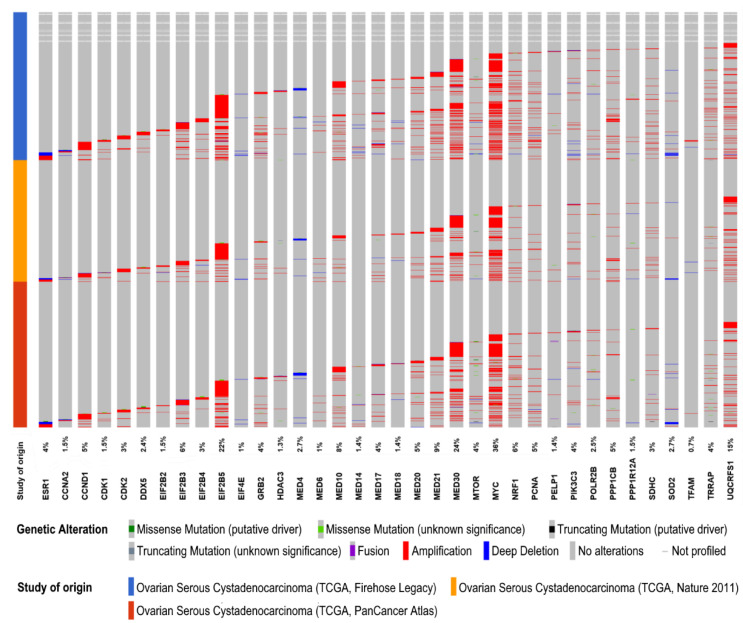
OncoPrint representation of the molecular landscape for ERα-associated fitness genes in ovarian cancer tissues collected in The Cancer Genome Atlas (TCGA), according to study of origin (Firehose Legacy, Nature 2011 and PanCancer Atlas). The OncoPrint provides an overview of genomic alterations (legend) per each gene across ovarian cancer cohorts (rows). ERα-associated fitness genes were altered in 74% of OC samples.

Estrogen-receptor pathway associated fitness genes in OC cells were altered in 74% of OC patient tissues and, noteworthy, most frequently amplified than deleted or mutated (Figure 5).

In order to extrapolate genes positively or negatively regulated by ESR1 in OC tissues, we performed a correlation analysis between ESR1 and the 35 fitness genes involved in the ERα signaling. The analysis was performed by comparing gene expression data from the RNA-Seq datasets collected by TCGA in Ovarian Serous Cystadenocarcinoma cohorts with the aid of cBioPortal tool [132,133,134].

As a result, we observed a statistically significant positive Spearman’s correlation (*p* value < 0.05) between ESR1 and MYC, an estrogen-responsive gene whose overexpression may contribute to acquired resistance in ER+ breast cancers [135]. This association is also supported by experimental evidences in OC cell lines, where estrogen treatment increases tumor burden and induces MYC expression [43]. Conversely, we observed a negative Spearman’s correlation between ESR1 and MED4, MED10, MED6 genes, encoding for three subunits of the Mediator complex. An ESR1 negative correlation was also detected with cell cycle regulators CCNA2 and CDK1 and the translation initiator factor eIF2B2.

Genome-scale CRISPR-Cas9 dropout screening in OC cell lines, combined with TCGA genomic and transcriptomic data, led us to hypothesize that ESR1 signaling might involve multiple interconnected pathways regulated by fitness genes in OC. This observation was further supported by experimental evidence of an ERα involvement in cancer cells proliferation and survival through the regulation of key-genes involved in cell cycle control, apoptosis, transcription, and through the activation of the MAPK signaling pathway, RAN signaling [75], activation of PI3K/AKT/mTOR, and Ras/MEK/ERK cascades [76,77].

### 5.5. Other Suitable Pathways for Targeted Therapies

Interestingly, among the multiple pathways influenced by ovarian cancer-related fitness genes, the mevalonate pathway, the telomere extension pathway, and different endocytic pathways were also present, each of them already known for its documented involvement in cancer physiology and development.

The mevalonate pathway is implicated in several key metabolic functions, leading to the production of essential sterol isoprenoids, like cholesterol, and non-sterol isoprenoids like dolichol, isopentenyl, and ubiquinone [136]. The rate-limiting enzyme of the mevalonate pathway is the hydroxymethyl-glutaryl coenzyme A (HMG-CoA) reductase (HMGCR), which converts HMG-CoA to mevalonic acid. From mevalonate the dimethylallyl pyrophosphate is then produced, that can in turn be condensed into either farnesyl pyrophosphate (FPP) and geranylgeranyl pyrophosphate (GGPP), involved in the process of protein prenylation, a fundamental step to facilitate protein attachment to membranes. This process is particularly important for post-translational modifications of Ras, Rho, Rab, and Rac small GTPase family proteins and enhance their membrane localization [137] and it is well known that many of these proteins are established oncogenes, associated with ovarian cancer cell aggressiveness and so influencing disease outcome [138].

While the correlation between the expression of tumor-specific HMGCR and ovarian cancer outcome has been pinpointed [139], it appears evident that statins assumption positively impacts on reducing OC risk [140,141,142], by inhibiting different aspects of the cancerous phenotype [143] and synergizing with chemotherapeutic agents [144,145] to enhance cell death. Indeed, the use of mevalonate pathway antagonist lovastatin has shown significant efficacy in reducing the proliferation of ovarian cancer cells in mouse xenograft models, regulating the expression of several essential genes involved in DNA replication, Rho/PLC signaling, glycolysis, and cholesterol biosynthesis pathways [146]. In addition, simvastatin, a widely used HMGCR inhibitor, has exhibited the ability to induce cell death of metastatic OC cells in syngeneic mouse models, which undergo extensive genetic reprogramming and overexpress mevalonate pathway-associated genes, conferring them resistance to apoptosis [147]. In line with these findings, the use of mevalonate pathway inhibitors, and in particular inhibitors of farnesyltransferase and geranylgeranyltransferase, has displayed marked effects in suppressing ovarian tumor growth, likely inducing autophagy and increased susceptibility to chemotherapy [148]. Lastly, administration of the farnesyl diphosphate synthase inhibitor zelodronic acid, a bisphosphonate, can cooperate with pitavastatin to synergistically inhibit the growth of ovarian cancer cells and induce apoptosis, by altering the subcellular localization of small GTPases [149]. Altogether, the importance of the mevalonate pathway and its derivate metabolites in the pathogenesis and development of ovarian cancer has pointed out the utility of drug repositioning, namely the employment in cancer treatment of drugs generally used for other purposes, to broaden the range of available therapeutic options and possibly overcome chemoresistance.

Telomeres correspond to the terminal parts of chromosomes, and include DNA tandem repeats complexed with proteins, whose activity provides telomere elongation and prevents the DNA damage repair machinery to recognize chromosome ends as double-strand breaks [150]. In this context, a protective and regulatory role is exerted by the shelterin complex, also called telosome, composed by six proteins: TERF1, TERF2, POT1, RAP1, TINF2, and TPP1 [151]. Although telomeres tend to have different lengths in cancer, it appears that ovarian carcinoma cells specifically activate telomerase and maintain short stable telomeres in vitro and in vivo [152]. Moreover, it is not unusual to find telomere fusions in ovarian tumor tissues, also at early stages, suggesting that telomere dysfunction may be essential in the initiation and progression of the disease [153]. Anyway, to date, no specific correlation between OC-specific mortality and telomere length has been found [154], but different genetic variants in telomere-maintenance genes have been associated to OC risk [155], as well as shorter telomere length [156]. Interestingly, it has been observed that alkylating agents treatment of responsive OC cells can produce a downregulation of telomerase activity, a phenotype not replicable in resistant cells [157]. Since telomerase activity is generally higher in cancer cells [158], different strategies have been explored to make it a druggable target to impair cancer cell survival; for example, the relative low hTERT expression in normal cells make it an ideal candidate for immuno-targeting [159]. Additionally, anti-telomerase antisense oligonucleotides [160] and hTERC-targeting siRNAs [161] have provided the possibility for novel fascinating therapeutic approaches. Shelterin complex is also being evaluated for target treatments. Indeed, compounds able to disrupt the telomer–shelterin interaction and uncapped chromosome ends can produce selective cytotoxic effects in tumor cells [162,163].

Finally, endocytosis provides the main cellular mechanism to recycle protein components from cell membrane, internalize external molecules, and attenuate receptor signaling. While clathrin-mediated endocytosis represents the best studied system, other endocytic compartments, like caveolae, contribute to spatio-temporal activation of signaling molecules and constitute platforms for the assembly of signaling complexes linking the endocytic and signaling programs [164]. Endocytic pathways can be involved in cancer progression in different ways, by sustaining oncogenic receptor signaling, regulating cell fate determination, cell cycle, and apoptosis, and by orchestrating the signals essential for directed cell movements [164]. Intracellular transport and membrane traffic through the Golgi complex is instead regulated by to coatomer complex I (COPI)-coated vesicles [165], essential to ensure protein quality control and correct sorting. An intact COPI is also essential for productive autophagy, a process dually involved in tumor progression; COPI members are in fact overexpressed in several types of cancer, including OC, and are associated with poor prognosis. Inhibition of COPI member results in increased cell death and may represent a suitable therapeutic target [166,167].

## 6. Conclusions

**Figure 6:**
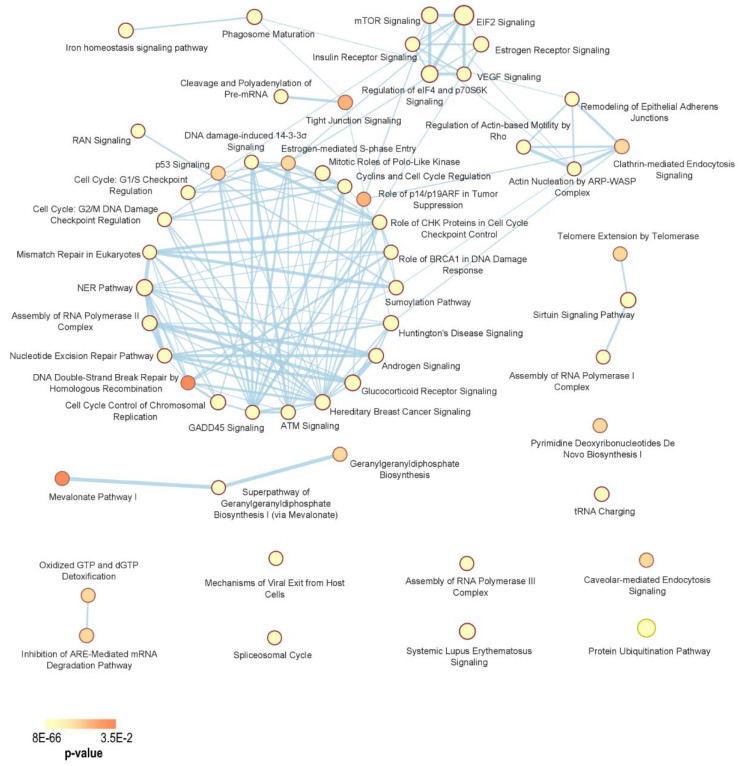
Canonical pathway enrichment analysis involving OC fitness genes using Ingenuity Pathway Analysis (IPA) and EnrichmentMap. Edges between nodes were generated using an overlap coefficient threshold of 0.3 and their width is proportional to the number of shared genes.

Ovarian cancers are among the most lethal and heterogeneous gynecological malignancies, with distinct clinicopathological and molecular features and prognosis, this representing a major challenge in their classification at both histological and molecular level. Indeed, inter- and intra-tumor heterogeneity seems to be the main cause of treatment failure. Molecular network changes are considered strong hallmarks of OC carcinogenesis and their exploitation an eligible tool for hub molecules discovery and the identification of targeted and personalized therapies. Loss of function screenings have recently emerged as promising approaches for the identification of candidate genes useful for the implementation of novel therapeutic protocols and possible drug repositioning in human cancers. Starting from the two most comprehensive CRISPR-Cas9 dropout screenings performed so far, we highlighted the most significantly affected functional pathways in OC. These involve 1213 genes that emerged as essential for cell viability and influencing more than 50 pathways relevant for the mainly characterized ovarian neoplasms (Figure 6). Most of them are interconnected with each other and some get more attention, such as the widely investigated DNA damage repair, VEGF, mTOR, EIF2, RAN, p53, ATM, iron homeostasis signaling and mevalonate pathway. Among them, the estrogen receptor signaling, although understudied mainly because of the challenging classification between ER-positive and ER-negative OCs and for discordant results of endocrine therapies, represents a traditional candidate gene which is emerging with an alternative look. Indeed, even though this receptor does not appear among the fitness genes for the OC cells considered, ER-related signaling pathways are strongly affected by several OC fitness genes. Moreover, ERα has been demonstrated to physically interact with most of these genes and considering TCGA patient-derived datasets, it results commonly mutated, in OC tissues, together with other ER-related essential genes. Thus, blocking estrogen signaling by targeting one or more of those ER-related genes could prove to be therapeutically effective.

## Figures and Tables

**Table 1:** Ovarian cancer cell lines, representative of different subtypes, used for cancer-related fitness genes identification.

Ovarian Cancer Subtype	Cell Lines
High Grade Serous	Caov-3, COV318, COV362, HEY A8, JHOS-2, JHOS-4, Kuramochi, OAW28, ONCO-DG-1, OV-90, OVCAR-8, Caov-4, HEY, OVCAR-5*, TYK-nu, OVCAR-3, OVMIU, PEO1, PEO4
Clear Cell	JHOC-5, OVISE, OVMANA, OVTOKO, ES-2, RMG-I, TOV-21G
Endometrioid	A2780, TOV-112D, A2780ADR, IGROV-1, OVK18, A2780cis
Mucinous	COV644, JHOM-1, RMUG-S, EFO-27, MCAS
Serous	SNU-8, UWB1.289, OAW42, OC 314, OVCA420
Mixed	59M, OV7
Brenner Tumor	SNU-840
Granulosa Cells Tumor	COV434
Unspecified	DOV13, EFO-21

* Ambiguous cell line: suspected to have an upper gastrointestinal origin [94].

**Table 2:** Canonical pathway analysis performed by Ingenuity Pathway Analysis (IPA) on ovarian cancer (OC) fitness genes.

Pathway	*p*-Value	Genes
**Cell cycle regulation and DNA damage response (DDR)**		
NER Pathway	1.58E−32	CCNH, CDK7, CHAF1A, CHAF1B, COPS2, COPS4, COPS5, COPS6, COPS8, DDB1, ERCC2, ERCC3, GPS1, GTF2H1, NEDD8, PCNA, POLA1, POLA2, POLD1, POLD2, POLD3, POLE, POLE2, POLR2B, POLR2C, POLR2D, POLR2E, POLR2F, POLR2G, POLR2H, POLR2I, POLR2K, POLR2L, PRIM1, RBX1, RFC2, RFC3, RFC4, RFC5, RPA1, RPA2, RPA3, TOP2A, UBE2I, UBE2N, USP7, XAB2
Cell Cycle Control of Chromosomal Replication	1.26E−22	CDC45, CDC6, CDC7, CDK1, CDK11A, CDK2, CDK7, CDK9, CDT1, DBF4, MCM2, MCM3, MCM4, MCM5, MCM6, MCM7, ORC1, ORC5, ORC6, PCNA, POLA1, POLA2, POLD1, POLE, PRIM1, RPA1, RPA2, RPA3, TOP2A
Mitotic Roles of Polo-Like Kinase	1.00E−17	ANAPC1, ANAPC10, ANAPC11, ANAPC2, ANAPC4, ANAPC5, CCNB1, CDC16, CDC20, CDC23, CDC26, CDC27, CDC7, CDK1, ESPL1, FBXO5, KIF11, KIF23, PKMYT1, PLK1, PLK4, PPP2R1A, PRC1, RAD21, SMC1A, SMC3, WEE1
Nucleotide Excision Repair Pathway	2.00E−14	CCNH, CDK7, ERCC2, ERCC3, GTF2H1, POLR2B, POLR2C, POLR2D, POLR2E, POLR2F, POLR2G, POLR2H, POLR2I, POLR2K, POLR2L, RPA1, RPA2, RPA3
Role of CHK Proteins in Cell Cycle Checkpoint Control	3.47E−09	ATR, CDK1, CDK2, CHEK1, CLSPN, HUS1, PCNA, PLK1, PPP2R1A, RAD1, RAD17, RAD9A, RFC2, RFC3, RFC4, RFC5, RPA1
DNA damage-induced 14-3-3σ Signaling	2.75E−06	ATR, CCNB1, CDK1, CDK2, HUS1, RAD1, RAD17, RAD9A
Mismatch Repair in Eukaryotes	8.71E−06	PCNA, POLD1, RFC2, RFC3, RFC4, RFC5, RPA1
Role of BRCA1 in DNA Damage Response	1.74E−05	ACTB, ATR, ATRIP, CHEK1, PLK1, RAD51, RBBP8, RFC2, RFC3, RFC4, RFC5, RPA1, SMARCB1, SMARCE1, TOPBP1
Cell Cycle: G2/M DNA Damage Checkpoint Regulation	4.07E−05	ATR, AURKA, CCNB1, CDK1, CDK7, CHEK1, PKMYT1, PLK1, SKP1, TOP2A, WEE1
ATM Signaling	4.90E−05	ATR, CCNB1, CDK1, CDK2, CHEK1, PPP2R1A, RAD17, RAD51, RAD9A, RBBP8, SMC1A, SMC2, SMC3, TOPBP1, TRRAP, USP7
Cell Cycle: G1/S Checkpoint Regulation	1.86E−04	ATR, CCND1, CDK2, GNL3, HDAC3, MAX, MYC, PAK1IP1, RPL11, RPL5, SIN3A, SKP1
Cyclins and Cell Cycle Regulation	3.24E−04	ATR, CCNA2, CCNB1, CCND1, CCNH, CDK1, CDK2, CDK7, HDAC3, PPP2R1A, SIN3A, SKP1, WEE1
Estrogen-mediated S-phase Entry	1.07E−02	CCNA2, CCND1, CDK1, CDK2, MYC
p53 Signaling	1.70E−02	ACTB, CDC42, CPSF1, CPSF2, CPSF3, CPSF6, CSTF3, GOSR2, NAPA, NAPG, NSF, NUDT21, PPP2R1A, RAC1, SYMPK, YKT6
Role of p14/p19ARF in Tumor Suppression	1.70E−02	NPM1, PIK3C3, RAC1, SF3A1, UBTF
DNA Double-Strand Break Repair by Homologous Recombination	3.47E−02	POLA1, RAD51, RPA1
**Hypoxia and Angiogenesis**		
EIF2 Signaling	7.94E−66	ACTB, CCND1, CDK11A, EIF1, EIF1AX, EIF2B2, EIF2B3, EIF2B4, EIF2B5, EIF2S1, EIF2S2, EIF2S3, EIF3A, EIF3B, EIF3D, EIF3E, EIF3F, EIF3G, EIF3I, EIF3M, EIF4A1, EIF4A3, EIF4E, EIF4G1, EIF5, FAU, GRB2, HSPA5, MYC, PABPC1, PDPK1, PIK3C3, PPP1CB, RPL10A, RPL11, RPL12, RPL13, RPL13A, RPL14, RPL15, RPL17, RPL18, RPL18A, RPL19, RPL23, RPL23A, RPL24, RPL26, RPL27, RPL27A, RPL28, RPL3, RPL30, RPL31, RPL32, RPL34, RPL35, RPL36, RPL37, RPL37A, RPL38, RPL4, RPL5, RPL6, RPL7, RPL7A, RPL7L1, RPL8, RPLP0, RPLP1, RPLP2, RPS11, RPS12, RPS13, RPS14, RPS15, RPS15A, RPS16, RPS18, RPS19, RPS2, RPS20, RPS21, RPS23, RPS24, RPS25, RPS27A, RPS28, RPS29, RPS3, RPS4X, RPS5, RPS6, RPS7, RPS8, RPS9, RPSA, UBA52, WARS1
Sirtuin Signaling Pathway	8.13E−05	GABPA, GTF3C2, MTOR, MYC, NDUFA11, NDUFAB1, NDUFB3, PAM16, POLR1A, POLR1B, POLR1C, POLR1E, POLR2F, RBBP8, RPTOR, RRP9, SDHC, SF3A1, SOD1, SOD2, TIMM10, TIMM13, TIMM23, TIMM44, TIMM9, TOMM22, TOMM40, TUBA1B, TUBA1C, UQCRFS1, XRCC5, XRCC6
VEGF Signaling	7.24E−04	ACTB, BCL2L1, EIF1, EIF1AX, EIF2B2, EIF2B3, EIF2B4, EIF2B5, EIF2S1, EIF2S2, EIF2S3, GRB2, PIK3C3, PTPN11
**Proliferative Signaling**		
Regulation of eIF4 and p70S6K Signaling	1.26E−29	EIF1, EIF1AX, EIF2B2, EIF2B3, EIF2B4, EIF2B5, EIF2S1, EIF2S2, EIF2S3, EIF3A, EIF3B, EIF3D, EIF3E, EIF3F, EIF3G, EIF3I, EIF3M, EIF4A1, EIF4A3, EIF4E, EIF4G1, FAU, GRB2, MTOR, PABPC1, PDPK1, PIK3C3, PPP2R1A, RPS11, RPS12, RPS13, RPS14, RPS15, RPS15A, RPS16, RPS18, RPS19, RPS2, RPS20, RPS21, RPS23, RPS24, RPS25, RPS27A, RPS28, RPS29, RPS3, RPS4X, RPS5, RPS6, RPS7, RPS8, RPS9, RPSA
mTOR Signaling	3.98E−18	CDC42, EIF3A, EIF3B, EIF3D, EIF3E, EIF3F, EIF3G, EIF3I, EIF3M, EIF4A1, EIF4A3, EIF4E, EIF4G1, FAU, GNB1L, MTOR, PDPK1, PIK3C3, PPP2R1A, RAC1, RHOQ, RPS11, RPS12, RPS13, RPS14, RPS15, RPS15A, RPS16, RPS18, RPS19, RPS2, RPS20, RPS21, RPS23, RPS24, RPS25, RPS27A, RPS28, RPS29, RPS3, RPS4X, RPS5, RPS6, RPS7, RPS8, RPS9, RPSA, RPTOR
Hereditary Breast Cancer Signaling	7.94E−12	ACTB, ATR, CCNB1, CCND1, CDK1, CHEK1, HDAC3, NPM1, PIK3C3, POLR2B, POLR2C, POLR2D, POLR2E, POLR2F, POLR2G, POLR2H, POLR2I, POLR2K, POLR2L, RAD51, RFC2, RFC3, RFC4, RFC5, RPA1, RPS27A, SMARCB1, SMARCE1, TUBG1, UBA52, WEE1
Iron homeostasis signaling pathway	2.24E−07	ACO2, ATP6AP1, ATP6V0B, ATP6V0C, ATP6V0D1, ATP6V1A, ATP6V1B2, ATP6V1C1, ATP6V1D, ATP6V1E1, ATP6V1F, ATP6V1G1, ATP6V1H, CIAO1, HSCB, HSPA9, ISCU, LYRM4, MMS19, NFS1, NUBP1, NUBP2, PCBP1, SKP1
Androgen Signaling	7.59E−07	CCND1, CCNH, CDK7, ERCC2, ERCC3, GNB1L, GTF2A1, GTF2B, GTF2E1, GTF2E2, GTF2F1, GTF2H1, POLR2B, POLR2C, POLR2D, POLR2E, POLR2F, POLR2G, POLR2H, POLR2I, POLR2K, POLR2L, TAF2
Glucocorticoid Receptor Signaling	1.58E−06	ACTB, BCL2L1, CCNH, CDK7, ERCC2, ERCC3, GRB2, GTF2A1, GTF2A2, GTF2B, GTF2E1, GTF2E2, GTF2F1, GTF2F2, GTF2H1, HSPA5, HSPA9, MED14, PIK3C3, POLR2B, POLR2C, POLR2D, POLR2E, POLR2F, POLR2G, POLR2H, POLR2I, POLR2K, POLR2L, RAC1, SMARCB1, SMARCE1, TAF1, TAF10, TAF12, TAF2, TAF6, TAF7, TSG101, UBE2I
RAN Signaling	1.62E−04	CSE1L, KPNB1, RAN, RANGAP1, RCC1, XPO1
Insulin Receptor Signaling	4.47E−04	CRKL, EIF2B2, EIF2B3, EIF2B4, EIF2B5, EIF4E, GRB2, MTOR, PDPK1, PIK3C3, PPP1CB, PPP1R10, PPP1R11, PPP1R12A, PPP1R7, PTPN11, RHOQ, RPTOR
Estrogen Receptor Signaling	6.92E−04	CCND1, DDX5, EIF2B2, EIF2B3, EIF2B4, EIF2B5, EIF4E, GRB2, HDAC3, MED10, MED14, MED17, MED18, MED20, MED21, MED30, MED4, MED6, MTOR, MYC, NRF1, PCNA, PELP1, PIK3C3, POLR2B, PPP1CB, PPP1R12A, SDHC, SOD2, TFAM, TRRAP, UQCRFS1
**Translation and post-translational modifications**		
Protein Ubiquitination Pathway	2.51E−24	ANAPC1, ANAPC10, ANAPC11, ANAPC2, ANAPC4, ANAPC5, BAP1, CDC20, CDC23, DNAJC17, DNAJC8, DNAJC9, HSCB, HSPA5, HSPA9, HSPD1, HSPE1, MED20, PSMA1, PSMA2, PSMA3, PSMA4, PSMA5, PSMA6, PSMA7, PSMB1, PSMB2, PSMB3, PSMB4, PSMB5, PSMB6, PSMB7, PSMC1, PSMC2, PSMC3, PSMC4, PSMC6, PSMD1, PSMD11, PSMD12, PSMD13, PSMD14, PSMD2, PSMD3, PSMD4, PSMD6, PSMD7, RBX1, RPS27A, SKP1, UBA1, UBA52, UBE2D3, UBE2I, UBE2L3, UBE2M, UBE2N, USP10, USP36, USP37, USP39, USP5, USP7, USP8
Assembly of RNA Polymerase II Complex	3.98E−23	CCNH, CDK7, DR1, ERCC2, ERCC3, GTF2A1, GTF2A2, GTF2B, GTF2E1, GTF2E2, GTF2F1, GTF2H1, POLR2B, POLR2C, POLR2D, POLR2E, POLR2F, POLR2G, POLR2H, POLR2I, POLR2K, POLR2L, TAF1, TAF10, TAF12, TAF2, TAF6, TAF7
tRNA Charging	3.16E−17	AARS1, CARS1, DARS1, EPRS1, FARSA, FARSB, GARS1, HARS1, IARS1, IARS2, KARS1, LARS1, MARS1, MARS2, NARS1, RARS1, SARS1, TARS1, VARS, WARS1, YARS1
Cleavage and Polyadenylation of Pre-mRNA	2.51E−08	CPSF1, CPSF2, CPSF3, CPSF6, CSTF3, NUDT21, PABPN1, WDR33
Assembly of RNA Polymerase I Complex	2.51E−08	POLR1A, POLR1B, POLR1C, POLR1E, POLR2F, TAF1B, TAF1C, UBTF
Assembly of RNA Polymerase III Complex	6.17E−08	BRF1, BRF2, GTF3A, GTF3C1, GTF3C2, GTF3C4, GTF3C5, SF3A1
Sumoylation Pathway	2.82E−05	CDC42, PCNA, RAC1, RAN, RANGAP1, RCC1, RFC2, RFC3, RFC4, RFC5, RHOQ, RNF4, RPA1, SAE1, SENP6, UBA2, UBE2I
Spliceosomal Cycle	2.82E−03	U2AF1/U2AF1L5, U2AF2
**Others**		
Systemic Lupus Erythematosus Signaling	1.58E−12	EFTUD2, GRB2, HNRNPC, LSM11, LSM2, LSM3, LSM4, LSM5, LSM6, LSM7, MTOR, PIK3C3, PRPF18, PRPF19, PRPF3, PRPF31, PRPF38A, PRPF38B, PRPF4, PRPF40A, PRPF4B, PRPF6, PRPF8, RNPC3, SART1, SF3B4, SNRNP200, SNRNP25, SNRNP27, SNRNP35, SNRNP40, SNRNP70, SNRPA1, SNRPB, SNRPD1, SNRPD2, SNRPD3, SNRPE, SNRPF, SNRPG, TXNL4A, ZMAT5
Phagosome Maturation	1.41E−09	ATP6AP1, ATP6V0B, ATP6V0C, ATP6V0D1, ATP6V1A, ATP6V1B2, ATP6V1C1, ATP6V1D, ATP6V1E1, ATP6V1F, ATP6V1G1, ATP6V1H, DYNC1H1, DYNC1I2, DYNLRB1, GOSR2, NAPA, NAPG, NSF, PIK3C3, TSG101, TUBA1B, TUBA1C, TUBB, TUBG1, VPS18, VPS28, VPS37A, YKT6
Huntington’s Disease Signaling	3.39E−06	BCL2L1, CLTC, DNM1L, DNM2, DYNC1I2, GNB1L, GOSR2, GRB2, HDAC3, HSPA5, HSPA9, MTOR, NAPA, NAPG, NSF, PDPK1, PIK3C3, POLR2B, POLR2C, POLR2D, POLR2E, POLR2F, POLR2G, POLR2H, POLR2I, POLR2K, POLR2L, RPS27A, SIN3A, UBA52, YKT6
Mechanisms of Viral Exit from Host Cells	4.27E−05	ACTB, CHMP2A, CHMP3, CHMP4B, CHMP6, SNF8, TSG101, VPS25, VPS28, XPO1
Remodeling of Epithelial Adherens Junctions	5.13E−05	ACTB, ACTR2, ACTR3, ARPC2, ARPC3, ARPC4, DNM1L, DNM2, HGS, TUBA1B, TUBA1C, TUBB, TUBG1
Superpathway of Geranylgeranyldiphosphate Biosynthesis I (via Mevalonate)	2.00E−03	FNTB, GGPS1, HMGCR, HMGCS1, MVK
Regulation of Actin-based Motility by Rho	4.07E−03	ACTB, ACTR2, ACTR3, ARPC2, ARPC3, ARPC4, CDC42, PFN1, PPP1CB, PPP1R12A, RAC1, RHOQ
Actin Nucleation by ARP-WASP Complex	4.57E−03	ACTR2, ACTR3, ARPC2, ARPC3, ARPC4, CDC42, GRB2, PPP1R12A, RAC1, RHOQ
Caveolar-mediated Endocytosis Signaling	5.01E−03	ACTB, ARCN1, COPA, COPB1, COPB2, COPE, COPG1, COPZ1, DNM2, ITGAV
Pyrimidine Deoxyribonucleotides De Novo Biosynthesis I	5.13E−03	CMPK1, DTYMK, DUT, RRM1, RRM2
Inhibition of ARE-Mediated mRNA Degradation Pathway	5.25E−03	CNOT1, CNOT3, DDX6, EXOSC2, EXOSC3, EXOSC4, EXOSC5, EXOSC6, EXOSC7, EXOSC8, EXOSC9, PABPN1, PPP2R1A, XRN1
Telomere Extension by Telomerase	6.76E−03	TERF1, TINF2, XRCC5, XRCC6
Clathrin-mediated Endocytosis Signaling	7.08E−03	ACTB, ACTR2, ACTR3, ARPC2, ARPC3, ARPC4, CDC42, CLTC, CSNK2B, DNM1L, DNM2, GAK, GRB2, HGS, PIK3C3, RAC1, RPS27A, TSG101, UBA52
Oxidized GTP and dGTP Detoxification	8.13E−03	DDX6, RUVBL2
Geranylgeranyldiphosphate Biosynthesis	8.13E−03	FNTB, GGPS1
Tight Junction Signaling	1.70E−02	ACTB, CDC42, CPSF1, CPSF2, CPSF3, CPSF6, CSTF3, GOSR2, NAPA, NAPG, NSF, NUDT21, PPP2R1A, RAC1, SYMPK, YKT6
Mevalonate Pathway I	3.47E−02	HMGCR, HMGCS1, MVK

Following, the fitness genes specifically involved in the most meaningful pathways representing the key hallmarks of OC will be summarized.

## Abbreviations

AKAP12	A-kinase anchor protein 12
AKT	Protein kinase B
APOBEC	Apolipoprotein B mRNA Editing Catalytic Polypeptide-like
ARID1A	AT-Rich Interaction Domain 1A
ATM	ATM serine/threonine kinase
ATR	ATR serine/threonine kinase
AURKA	Aurora kinase A
BARD1	BRCA1 associated RING domain 1
BAX	BCL2 associated X, apoptosis regulator
BCL2	BCL2 apoptosis regulator
BCL2L1	BCL2 like 1
BENE	Benzoate transport protein
Bit1	Bcl2-inhibitor of transcription 1
BRAF	B-Raf proto-oncogene, serine/threonine kinase
BRCA1	BReast CAncer gene 1
BRCA2	BReast CAncer gene 2
BRIP1	BRCA1 interacting protein C-terminal helicase 1
CASP4	Caspase 4
CCNA2	Cyclin-A2
CCNB1	Cyclin-B1
CCND1	Cyclin-D1
CCNE1	Cyclin-E1
CCOC	Clear cell ovarian cancer
CDC25	Cell division cycle 25 homolog A
CDH6	Cadherin-6
CDK	Cyclin-dependent kinases
CDK1	Cyclin-dependent kinases 1
CDK12	Cyclin-dependent kinases 12
CDK2	Cyclin-dependent kinases 2
CDKN2A	Cyclin-dependent kinase inhibitor 2A
CHEK1	Checkpoint kinase 1
CHEK2	Checkpoint kinase 2
COPI	COPI-coated vesicles
CREBBP	CREB-binding protein
CSE1L	Human homolog of the yeast cse1gene
CSMD3	CUB and Sushi multiple domains 3
CTNNB1	Catenin beta 1
CTSD	Cathepsin D
CXCL11	C-X-C motif chemokine 11
CXCR7	C-X-C Chemokine Receptor Type 7
CYR61	Cysteine rich angiogenic inducer 61
DDR	DNA damage response
DES	Desmin
DOT1L	Disruptor of telomeric silencing-1-like
DSB	Double-strand DNA breaks
DYNLL1	Dynein light chain LC8-type 1
E2	17β-estradiol
EIF2	Eukaryotic translation initiation factor 2
EIF2B1	Eukaryotic translation initiation factor 2B subunit alpha
EIF2B2	Eukaryotic translation initiation factor 2B subunit beta
EIF2B3	Eukaryotic translation initiation factor 2B subunit gamma
EIF2B4	Eukaryotic translation initiation factor 2B subunit delta
EIF2B5	Eukaryotic translation initiation factor 2B subunit epsilon
EIF4E	Eukaryotic translation initiation factor 4E
EOC	Epithelial ovaria cancer
EOVC	Endometrioid ovarian cancers
ERBB2	Erb-b2 receptor tyrosine kinase 2
ERCC	NA excision repair protein ERCC-1-like protein
ERCC2	ERCC excision repair 2
ERCC3	ERCC excision repair 3
ERE	Estrogen-response element
ERK	Extracellular regulated MAP kinase
ERα	Estrogen receptor α
ERβ	Estrogen receptor β
ESR1	Estrogen receptor 1
ESR2	Estrogen receptor 2
FAT3	FAT atypical cadherin 3
FOSL1	FOS like 1, AP-1 transcription factor subunit
FOXM1	Forkhead box M1
FPP	Farnesyl pyrophosphate
GABRA6	Gamma-aminobutyric acid type A receptor subunit alpha6
GGPP	Geranylgeranyl pyrophosphate
GO	Gene Ontology
GRSF1	G-rich RNA sequence binding factor 1
HDAC3	Histone deacetylase 3
HDI	Human Development Index
HER2	Human epidermal growth factor receptor 2
HGSOC	High-grade serous ovarian cancer
HMG-CoA	Hydroxymethyl-glutaryl coenzyme A
HMGCR	Hydroxymethyl-glutaryl reductase
HR	Homologous recombination
HRT	Hormone replacement therapy
hTER	Telomerase reverse transcriptase
HUS1	HUS1 checkpoint clamp component
ID4	Inhibitor of DNA binding 4, HLH protein
IGFBP3	Insulin like growth factor binding protein 3
IPA	Ingenuity Pathway Analysis
IRF2BP2	Interferon regulatory factor 2 binding protein 2
KMT2B	Lysine methyltransferase 2B
KMT2D	lysine methyltransferase 2D
KPNB1	Karyopherin subunit beta 1
KRAS	KRAS proto-oncogene, GTPase
KRT	Keratin
LCN2	Lipocalin-2
LGSOC	Low-grade serous ovarian cancer
LOH	Loss of heterozygosity
MAPK	Mitogen-Activated Protein Kinase
MECOM	MDS1 and EVI1 complex locus
MED	Mediator Complex
MED14	Mediator Complex Subunit 14
MED17	Mediator Complex Subunit 17
MED18	Mediator Complex Subunit 18
MED20	Mediator Complex Subunit 20
MED21	Mediator Complex Subunit 21
MED30	Mediator Complex Subunit 30
MED4	Mediator Complex Subunit 4
MED6	Mediator Complex Subunit 6
MLH1	MutL homolog 1
MMP	Matrix metalloproteinase
MMR	DNA mismatch repair
MOC	Mucinous ovarian cancers
Mre11	MRE11 homolog, double strand break repair nuclease
MTOR	Mammalian target of rapamycin
MTORC1	Mammalian target of rapamycin complex 1
MUTYH	MutY DNA glycosylase
MYC	MYC proto-oncogene
NBS1	Nijmegen Breakage Syndrome 1
NER	Nucleotide excision repair
NF1	Neurofibromin 1
NOTCH4	Notch Receptor 4
NRAS	NRAS proto-oncogene, GTPase
OC	Ovarian cancer
P16	Cyclin-dependent kinase inhibitor 2A
P21	Cyclin-dependent kinase inhibitor 1
P27	Cyclin-dependent kinase inhibitor 1B
PALB2	Partner and localizer of BRCA2
PARP	Poly ADP ribose polymerase
PAX2	Paired box gene 2
PAX8	Paired box gene 8
PCNA	Proliferating cell nuclear antigen
PERK	Protein kinase-like Endoplasmic Reticulum Kinase
PGR	Progesterone receptor
PIK3C3	Phosphatidylinositol 3-Kinase Catalytic Subunit Type 3
PI3K	Phosphatidylinositol 3-kinase
PI3KCA	Phosphatidylinositol 3-kinase catalytic alpha polypeptide
PLAU	Plasminogen activator, urokinase
PLC	Phospholipase C
PLK1	Polo like kinase 1
POLE	DNA polymerase epsilon, catalytic subunit
POT1	Protection of telomeres 1
PP2R1A	Protein phosphatase 2, regulatory subunit A
PPP1CB	Protein Phosphatase 1 Catalytic Subunit Beta
PPP1R12A	Protein Phosphatase 1 Regulatory Subunit 12A
PPP2R1A	Protein Phosphatase 2 Scaffold Subunit Aalpha
PTEN	Phosphatase and tensin homolog
Rab	Rab Family Small GTPase
Rac	Rac Family Small GTPase
RAD1	RAD1 checkpoint DNA exonuclease
RAD50	RAD50 double strand break repair protein
RAD51	RAD51 recombinase
RAD51C	RAD51 paralog C
RAD51D	RAD51 paralog D
RAD9	Checkpoint Clamp Component A
RAN	Ras-related nuclear protein
RAP1	Ras-related protein 1
RASSF1gene	Ras Association Domain Family Member 1
RB1	Retinoblastoma protein
RCF3	Replication factor C subunit 3
RFC	Replication factor C
Rho	Rhodopsin
RhoA	Ras homolog family member A
RPA	Replication Protein A
RPA3	Replication Protein A3
RRAS2	Ras-Related Protein R-Ras2
STK11	Serine/threonine kinase 11
TCGA	The Cancer Genome Atlas
TERF1	Telomeric Repeat Binding Factor 1
TERF2	Telomeric Repeat Binding Factor 2
TFAP4	Transcription Factor AP-4
TGFBI	Transforming Growth Factor Beta Induced
TIGAR	TP53 induced glycolysis and apoptosis regulator
TINF2	TERF1 Interacting Nuclear Factor 2
TNFSF7	Tumor Necrosis Factor Ligand Superfamily Member 7
TP53	Tumor protein p53
TPP1	Tripeptidyl peptidase 1
TRAM1	Translocation associated membrane protein 1
TRAP1	TNF receptor associated protein 1
UBL1	Ubiquitin-like protein 1
UPR	Unfolded protein response
VEGF	Vascular endothelial growth factor
VIM	Vimentin
WEE1	WEE1 G2 checkpoint kinase
ZMYND8	Zinc finger MYND-type containing 8
ZNF587B	Zinc finger protein 587B
